# Change of Title: From *High-Throughput* to *BioTech*

**DOI:** 10.3390/biotech9040018

**Published:** 2020-09-22

**Authors:** Paolo Iadarola, Massimo Negrini

**Affiliations:** 1 Department of Biology and Biotechnologies “L.Spallanzani”, University of Pavia, 27100 Pavia, Italy; paolo.iadarola@unipv.it; 2 Department of Morphology, Surgery and Experimental Medicine, University of Ferrara, 44121 Ferrara, Italy

Founded in 2012, *High-Throughput* (formerly *Microarrays*) is a MDPI peer-reviewed journal that has published 216 articles so far, 29 of which are frequently cited (10 to 100 times) reports.

The publication of experimental data in numerous fields (Chemistry, Biochemistry and Life Sciences in general), soon evidenced the multidisciplinary approach of the journal. The articles published over these years contain topics ranging from the presentation of computational and statistical tools for data analysis and interpretation [1,2], to the development of novel high-throughput techniques [3,4,5,6], the search for new materials [7,8], and a variety of results in the “omics” field [9,10,11,12,13,14]. If ever there was a doubt, the current publication of excellent works spanning these fields is the proof that the journal has successfully achieved its intended purpose.

Alongside with regular volumes, a good number of special issues led by guest editors who are experts in the subject have published collections of articles focused on specific topics.

The journal has now arrived at a turning point since *High-Throughput* will soon be renamed *BioTech* from the fourth issue in 2020. What does this change of name entail?

The biotech area is well known to encompass a wide range of fields and procedures including genomics, proteomics, applied immunology, recombinant gene techniques, and development of pharmaceutical therapies.

Of particular interest is medical biotechnology, with all its applications aimed at improving the health of human beings. Investigations on technologies used to treat heritable and nonheritable diseases, as well as the production of therapeutic human proteins, or the use of living cells are of interest for the journal. Additional topics include investigations that help to optimize the use of blood as a source diagnostic, prognostic, or predictive information in human diseases, or the use of innovative diagnostic approaches for the early detection of cancer. 

*BioTech* journal is interested in publishing papers in the genetics of plants and farm animals that can lead to an improvement in production and resistance to adverse climates or parasites.

*BioTech* has the main objective of being a vehicle for publications whose purpose is to improve the conditions of human beings. In so doing, *BioTech* will promote the publication of articles that show how modern technologies can have a positive impact in medicine as well as the production of food, pharmaceuticals, chemical products, and energy sources.

On behalf of the Editorial Board and the Editorial Office, we wish to thank all authors, reviewers and external editors for their support to *High-Throughput* during these years and welcome them to join us in developing *BioTech* to its full potential.
